# Doping-induced memory effect in Li-ion batteries: the case of Al-doped Li_4_Ti_5_O_12_†
†Electronic Supplementary Information (ESI) available: XRD, SEM and EIS measurements, and other related information. See DOI: 10.1039/c5sc00429b


**DOI:** 10.1039/c5sc00429b

**Published:** 2015-04-17

**Authors:** De Li, Yang Sun, Xizheng Liu, Ruwen Peng, Haoshen Zhou

**Affiliations:** a Energy Technology Research Institute , National Institute of Advanced Industrial Science and Technology (AIST) , Umezono, 1-1-1 , Tsukuba , 305-8568 , Japan . Email: hs.zhou@aist.go.jp ; Fax: +81-29-861-3489; b National Laboratory of Solid State Microstructures & Department of Energy Science and Engineering , Nanjing University , Nanjing 210093 , China; c National Laboratory of Solid State Microstructures & Department of Physics , Nanjing University , Nanjing 210093 , China

## Abstract

In Li-ion batteries (LIBs), a memory effect has been revealed in two-phase electrode materials such as olivine LiFePO_4_ and anatase TiO_2_, which complicates the two-phase transition and influences the estimation of the state of charge. Practical electrode materials are usually optimized by the element doping strategy, however, its impact on the memory effect has not been reported yet. Here we firstly present the doping-induced memory effect in LIBs. Pristine Li_4_Ti_5_O_12_ is free from the memory effect, while a distinct memory effect could be induced by Al-doping. After being discharged to a lower cutoff potential, Al-doped Li_4_Ti_5_O_12_ exhibits poorer electrochemical kinetics, delivering a larger overpotential during the charging process. This dependence of the overpotential on the discharging cutoff leads to the memory effect in Al-doped Li_4_Ti_5_O_12_. Our discovery emphasizes the impact of element doping on the memory effect of electrode materials, and thus has implications for battery design.

## Abstract 2

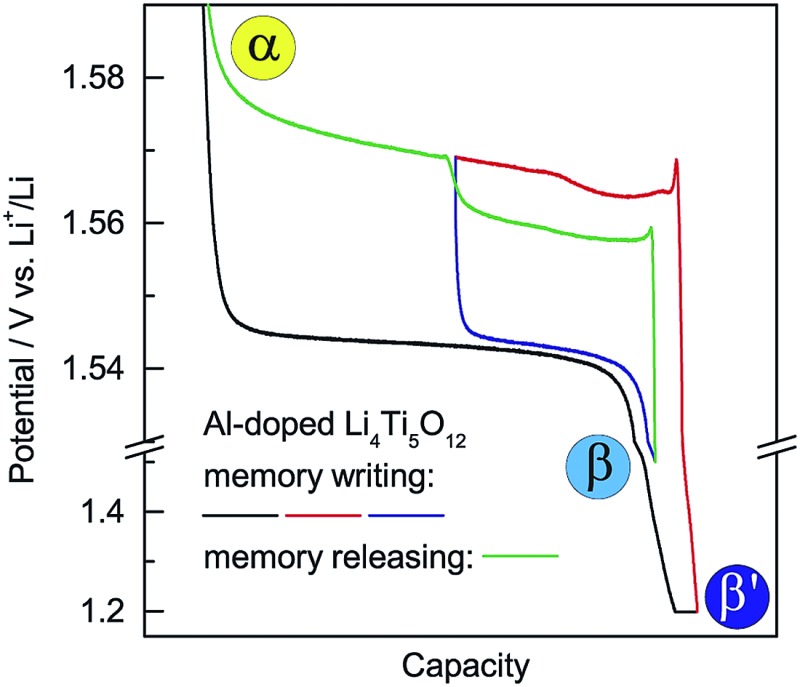
A memory effect in Li-ion batteries can be induced and tailored by element doping, such as Al-doping in spinel Li_4_Ti_5_O_12_.

## Introduction

Li-ion batteries (LIBs) are the state-of-the-art power source for mobile electrical devices, electric vehicles and smart grids.^1^ It was generally accepted that LIBs are free from the memory effect,^2^ which is common in nickel–cadmium (Ni–Cd) and nickel-metal-hydride (Ni-MH) batteries.^3^–^5^ However, recent studies indicated that the memory effect also exists in olivine LiFePO_4_ and anatase TiO_2_ in LIBs.^6^,^7^ As both LiFePO_4_ (ref. 8 and 9) and TiO_2_ (ref. 10) undergo a two-phase reaction upon charge/discharge,^11^–^13^ which leads to a very flat potential plateau, even minimal potential changes from the memory effect will make it difficult to estimate the state of charge (SOC) in LIBs.

The memory effect in an olivine LiFePO_4_ cathode was firstly reported by Sasaki *et al.*,^6^ and was rationalized by using the particle-by-particle model.^14^ In the memory-writing cycle, LiFePO_4_ particles are divided into two groups: some of the LiFePO_4_ particles (first group) undergo an extra charge/discharge cycle relative to the others (second group). In the memory-releasing cycle, the second group is (dis)charged with a larger overpotential compared with the first group, which results in a potential bump in the (dis)charging curve. The memory effect is closely associated with the overshooting phenomenon at the beginning of (dis)charging.^6^,^7^ Owing to a much smaller initial overshoot, no memory effect has been observed in spinel Li_4_Ti_5_O_12_.^6^

Practical electrode materials are usually optimized by the element doping strategy,^15^ while its impact on the memory effect remains unknown. In this paper, we try to examine the impact of a doping strategy, and we focus on spinel Li_4_Ti_5_O_12_,^16^ which is a renowned long-cycle-life anode material for LIBs.^17^,^18^ Although pristine Li_4_Ti_5_O_12_ is free from the memory effect, we find that a distinct memory effect could be induced by Al-doping.

## Experimental

Pristine Li_4_Ti_5_O_12_ (LTO) was provided by Ishihara Sangyo Kaisha, Ltd. and nano-Al_2_O_3_ (mean particle size: 22.2–47.7 nm) was supplied by Nanophase Technologies Corp. (NanoTek®). LTO and nano-Al_2_O_3_ (10 wt%) were ground thoroughly and the mixture was calcined at 800 °C for 24 h in a vacuum to obtain Al-doped Li_4_Ti_5_O_12_ (ALTO). The morphologies and crystal structures were characterized by scanning electron microscopy (SEM, LEO Gemini Supra 35) and powder X-ray diffraction (XRD, Cu Kα radiation, Bruker D8 Advance Diffractometer), respectively. The galvanostatic and voltammetric measurements were performed at room temperature with a battery charge/discharge system from Hokuto Denko Corp. and an Autolab electrochemical instrument, respectively.

Electrochemical measurements were conducted using coin cells (CR2032). In the working electrode, a composite paste, containing 42.5 wt% LTO or ALTO, 42.5 wt% acetylene black and 15 wt% polytetrafluoroethylene (PTFE), was firmly pressed on an Al mesh (100 mesh) with a mass loading of *ca.* 4 mg cm^–2^. Here, 42.5 wt% acetylene black was added to enhance the electronic conductivity. The counter electrode of lithium metal was separated from the working electrode by a Celgard 2400 porous polypropylene film, and the electrolyte used was 1 M LiClO_4_ in ethylene carbonate/diethyl carbonate (EC/DEC with a volume ratio of 1 : 1). After drying all components, the cells were assembled in a glovebox filled with argon gas.

## Results and discussion

Fig. S1a† shows X-ray diffraction (XRD) patterns of pristine Li_4_Ti_5_O_12_ (LTO) and Al-doped Li_4_Ti_5_O_12_ (ALTO). The XRD pattern of ALTO can be well indexed with the spinel structure, indicating no evident structural change after Al-doping, although a minor amount of Al_2_O_3_ and rutile TiO_2_ impurities exist. The peaks of ALTO shift to higher angles compared with those of LTO (Fig. S1b†), suggesting decreased lattice parameters, which is consistent with previous reports.^19^,^20^ Besides, both of the samples are assemblies of nano-crystallites (Fig. S2a and b†).

**Fig. 1:**
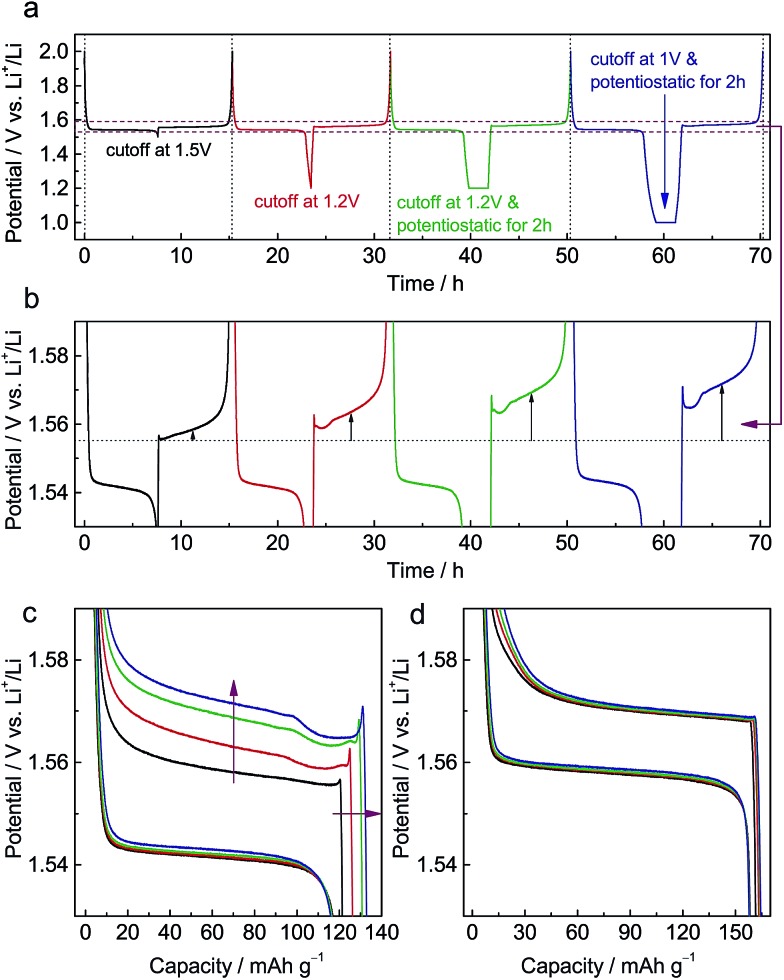
Electrochemical dependence on the discharging cutoff in Al-doped Li_4_Ti_5_O_12_ (ALTO). (a) A sequence of four cycles: (1) discharge to 1.5 V and full charge; (2) discharge to 1.2 V and full charge; (3) discharge to 1.2 V and potentiostatic for 2 h, and full charge; (4) discharge to 1.0 V and potentiostatic for 2 h, and full charge. (b) Enlarged view between 1.53 and 1.59 V. (c) The charge/discharge curves in these four cycles. (d) The charge/discharge curves in the same four cycles of pristine Li_4_Ti_5_O_12_ (LTO). The current rate is 0.1 C.

Galvanostatic measurements were performed on ALTO in a sequence of four cycles with different discharging cutoffs (Fig. 1a). As the cutoff potential decreases, especially when potentiostatic for 2 h, the subsequent charging potential heightens (Fig. 1b). A previous report has also indicated that the charging potential of Al-doped Li_4_Ti_5_O_12_ heightens for the low discharging cutoff.^19^ As the cutoff becomes lower, the capacity of subsequent charging is also increased (Fig. 1c). In the charging curves, a distinct overshoot appears at the beginning, the height of which is also dependent on the cutoff. For comparison, LTO was also measured following the same procedure, while the charging curves show almost no evident dependence on the discharging cutoff (Fig. 1d). Compared with LTO, the relatively low specific capacity of ALTO could be attributed to the impurities and Al-doping. Besides, the discharging potential of ALTO is lower than that of LTO, indicating that the Al-doping raises the energy of the Ti^4+^/Ti^3+^ redox couple in spinel Li_4_Ti_5_O_12_.^21^

We also examined the electrochemical dependence on the charging cutoff for ALTO (Fig. S3a†). The results show that the discharging curves are independent of the charging cutoff (Fig. S3b†). For different charging cutoffs, the subsequent discharging curves are nearly overlapped between these three cycles. Note that there is no overshoot at the beginning of discharging (Fig. S3c†), consistent with the association between the memory effect and initial overshoot. For LTO, analogous results are obtained (Fig. S3d†).

**Fig. 2:**
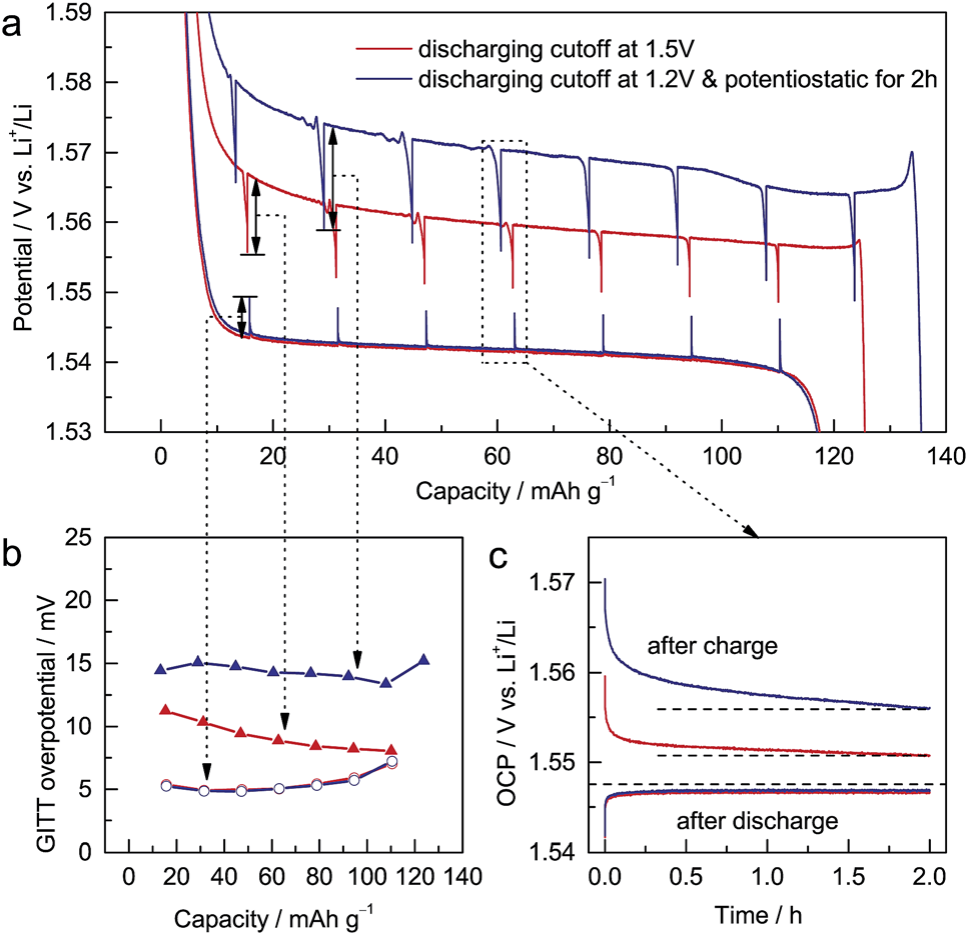
(a) GITT measurements for Al-doped Li_4_Ti_5_O_12_ (ALTO), which consist of a series of current pulses applied at 0.1 C for 1 h, each followed by a 2 h relaxation period. The charging cutoff is 2.0 V, and the discharging cutoffs were 1.5 V (red) and 1.2 V and potentiostatic for 2 h (blue). (b) Potential increments (decrements) and (c) OCPs during the relaxation periods.

To study the electrochemical differences caused by different discharging cutoffs, galvanostatic intermittent titration technique (GITT) measurements^22^ were performed on ALTO. For different cutoffs, the subsequent charging curves vary significantly (Fig. 2a). The potential decrement during relaxation in charging is also dependent on the cutoff: a lower cutoff leads to a larger potential decrement (Fig. 2b). Compared with the potential increments in discharging, the potential decrements in charging are much larger. During the relaxation in discharging, the open-circuit potential (OCP) almost approaches the equilibrium potential in two hours (Fig. 2c). In contrast, the OCP in charging continuously decreases as a tilted line, which is more evident for the lower cutoff. These phenomena indicate that the kinetics of the charging process, which are inferior to those of the discharging process, are dependent on the discharging cutoff.

**Fig. 3:**
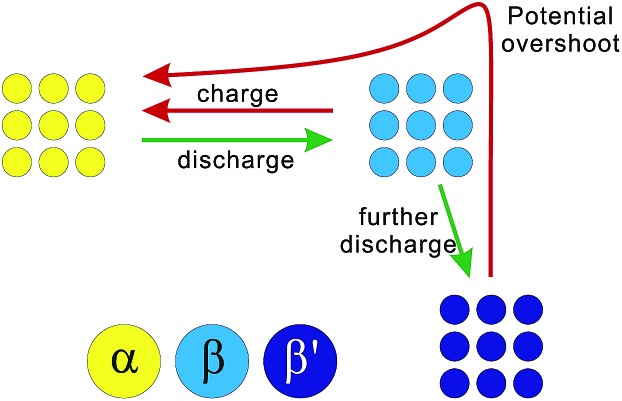
Schematic diagram of phase transitions in Al-doped Li_4_Ti_5_O_12_ under different discharging cutoffs, in which α is the delithiated phase, β is the lithiated phase, and β′ is the lithiated phase discharged to a very low cutoff potential.

According to the above results and analysis, we may infer that the status of the ALTO electrode depends on the discharging cutoff, different to the undoped LTO. Fig. 1c and d show that the specific capacity of ALTO increases with a decreased discharging cutoff, while it almost remains constant in LTO. This indicates that a high discharging cutoff is enough to achieve the full lithiation of LTO. For ALTO however, a few of the Li sites are difficult to access and require a low discharging cutoff to realize the Li-ion insertion. These “hardly accessible” Li sites could be reasonably attributed to the Al-doping induced local structural change, while the detailed lattice structure after doping is still unknown and needs more investigation. Fig. 3 schematically illustrates the phase transition behaviour of ALTO with different discharging cutoffs. In the case of a high cutoff, the phase transition between the α (delithiated) and β (lithiated) phases could occur readily. With further discharging to a low cutoff, the β phase will be transformed into the β′ phase, which represents the lithiated phase after the deeper discharging, relative to the β phase. In the subsequent charging process, a high potential is necessary to convert the β′ phase into the α phase, owing to its poor electrochemical kinetics. The poor kinetics of the β′ phase could also be reflected in the high initial overshoot occurring during the charging process.

To identify the difference between the β and β′ phases, the discharged ALTO with different discharging cutoffs was characterized using electrochemical impedance spectroscopy (EIS). The electrode was firstly discharged to different cutoff potentials and subsequently charged to 1.5 V, then the corresponding EIS spectra were measured from 10^6^ Hz to 10^–3^ Hz (Fig. S4†). In the high frequency region, the depressed semicircle (the inset in Fig. S4b†) is associated with the charge-transfer resistance (*R*_ct_), which barely changes for different discharging cutoffs. In the low frequency region, the bounded-diffusion impedance exhibits a transition from the Warburg regime to the capacitive regime by decreasing the frequency (Fig. S4b†),^23^ and it enlarges evidently for the lower discharging cutoff (Fig. S4b and c†). In other words, the diffusion impedance of the β′ phase is larger than that of β phase for discharged ALTO, validating the difference in their electrochemical kinetics. In contrast, no evident difference was found in the EIS spectra of LTO with different discharging cutoffs (Fig. S5†).

**Fig. 4:**
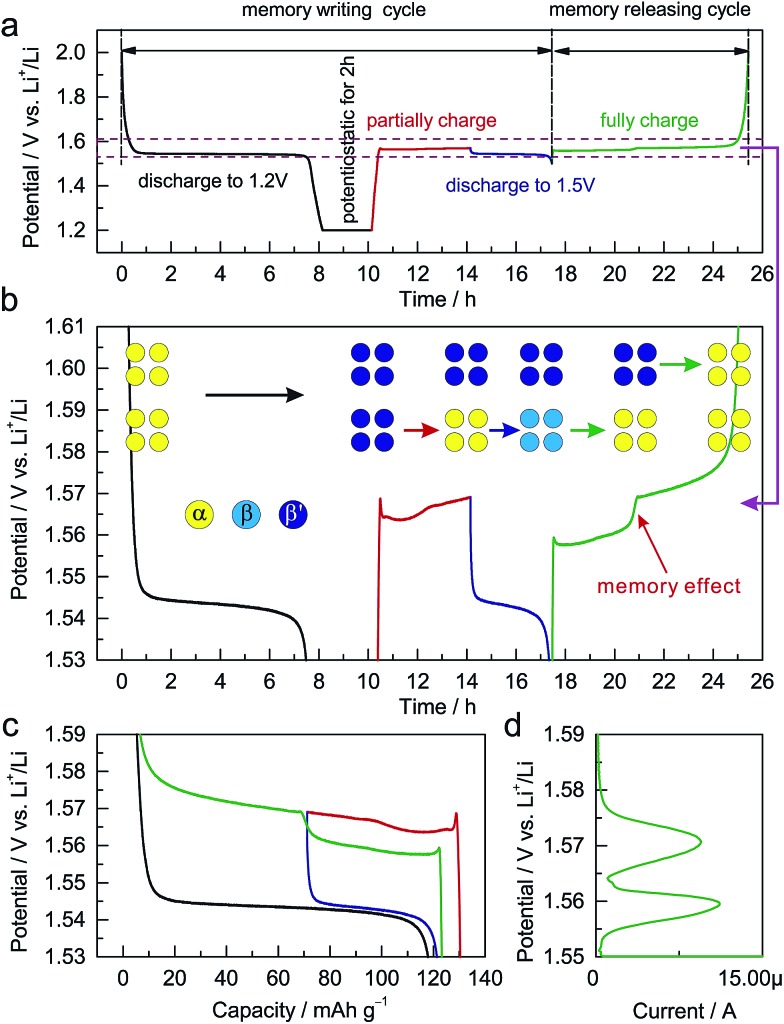
Demonstration of a memory effect in Al-doped Li_4_Ti_5_O_12_ (ALTO). (a) Memory-writing cycle: discharge to 1.2 V and potentiostatic for 2 h (black), partially charged for 4 h (red), and discharge to 1.5 V (blue); memory-releasing cycle: full charge to 2.0 V (green). The current rate is 0.1 C. (b) Enlarged view between 1.53 and 1.61 V. The inset shows the corresponding phase transition, in which α is the delithiated phase, β is the lithiated phase discharged to 1.5 V, and β′ is the lithiated phase discharged to 1.2 V and potentiostatic for 2 h. (c) The charge/discharge curves in these memory-writing/releasing cycles. (d) The linear sweep voltammetry (LSV) curve of ALTO with a scan rate of 1 μV s^–1^ from 1.55 to 1.59 V after the same memory-writing cycle.

The dependence of the charging potential on the preceding discharging cutoff is equivalent to a memory effect in LIBs. Analogous to the reported memory effect in olivine LiFePO_4_ and anatase TiO_2_,^6^,^7^ ALTO exhibits a two-step charging curve after a special memory-writing cycle, which is a typical memory effect (Fig. 4a and b). The corresponding phase transition can be described by a schematic model (Fig. 4b). Firstly, all particles in the electrode are discharged to 1.2 V and are potentiostatic for 2 h; secondly, a group of particles (lower) are charged and then discharged to 1.5 V, while the other group (upper) do not change; then, the lower group with the discharging cutoff of 1.5 V is charged first at a low potential, followed by the charging of the upper group at a high potential. Although the charging process from the low discharging cutoff is interrupted by a partial discharge/charge cycle (blue curve and the lower step of the green curve), the charging curve can still be smoothly connected as the red curve and the higher step of the green curve (Fig. 4c). The memory effect can also be reflected in the linear sweep voltammetry (LSV) result (Fig. 4d). The two peaks in the LSV mode correspond to the two steps in the galvanostatic mode. These results unambiguously demonstrate the memory effect in the electrochemical processes of ALTO. By comparison, no memory effect has been observed in LTO (Fig. S6†).

**Fig. 5:**
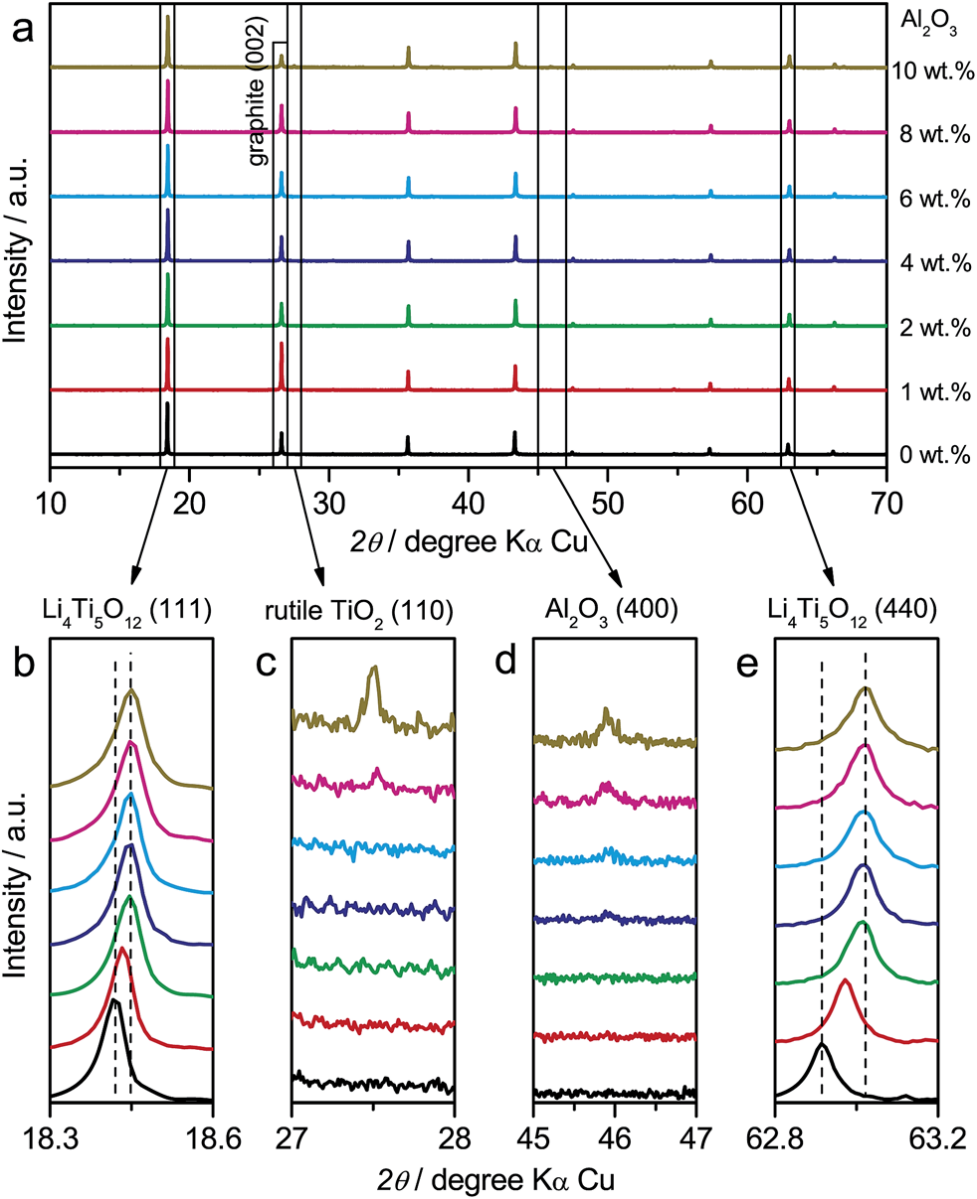
(a) XRD patterns of Al-doped Li_4_Ti_5_O_12_ with different doping levels. In the precursor, the amount of Al_2_O_3_ is varied from 0 wt% to 10 wt%. Enlarged XRD patterns of (b) Li_4_Ti_5_O_12_ (111), (c) rutile TiO_2_ (110), (d) Al_2_O_3_ (400), (e) Li_4_Ti_5_O_12_ (440) peaks. During the XRD measurements, *ca.* 5% graphite is mixed into the samples to calibrate the position of peaks.

In addition to LTO and ALTO described above, we also studied a series of Al-doped Li_4_Ti_5_O_12_ with different doping levels. The amount of nano-Al_2_O_3_ was varied from 0 wt% to 10 wt% in the precursor to change the doping level of Al-doped Li_4_Ti_5_O_12_. The precursor of pristine Li_4_Ti_5_O_12_ (Sigma-Aldrich Co. LLC.) and nano-Al_2_O_3_ mixture was ground thoroughly and calcined at 800 °C for 24 h in air to obtain Al-doped Li_4_Ti_5_O_12_. Fig. 5a shows the XRD patterns of Al-doped Li_4_Ti_5_O_12_ with different doping levels. The XRD peaks of Al-doped Li_4_Ti_5_O_12_ shift towards higher angles as the amount of Al_2_O_3_ increases from 0 wt% to 2 wt%. Above 2 wt%, the peaks of identical index approach a constant angle (Fig. 5b and e). Rutile TiO_2_ appears when the Al_2_O_3_ amount is over 8 wt% (Fig. 5c), and the amount of Al_2_O_3_ residue increases as the amount of Al_2_O_3_ precursor is varied from 0 wt% to 10 wt% (Fig. 5d). The above results indicate that high-level Al-doping will lead to phase separation associated with the formation of rutile TiO_2_.^24^

**Fig. 6:**
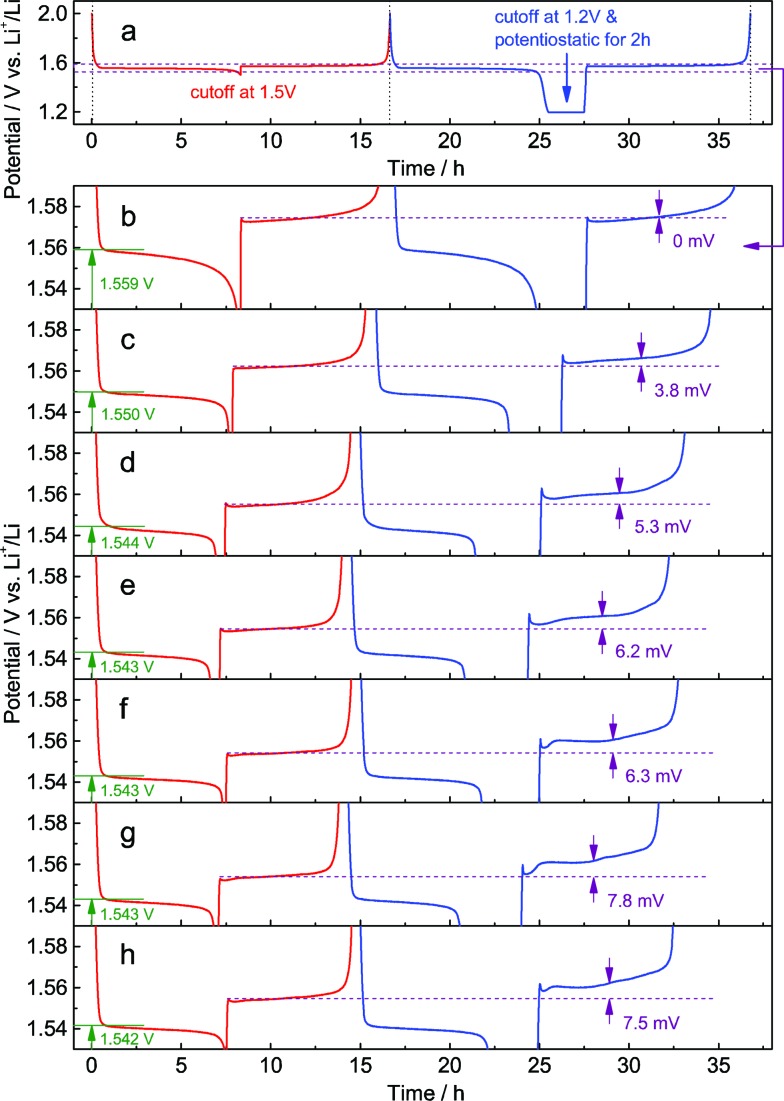
Electrochemical dependence of Al-doped Li_4_Ti_5_O_12_ on the doping level. (a) The sequence of two cycles. 1st cycle: discharge to 1.5 V and full charge; 2nd cycle: discharge to 1.2 V and potentiostatic for 2 h, and full charge. Enlarged view between 1.53 and 1.59 V for Al-doped Li_4_Ti_5_O_12_, the precursor of which contains (b) 0 wt%, (c) 1 wt%, (d) 2 wt%, (e) 4 wt%, (f) 6 wt%, (g) 8 wt%, and (h) 10 wt% Al_2_O_3_. The charge/discharge current rate is 0.1 C.

In the galvanostatic measurements, a sequence of two cycles with different discharging cutoffs was performed on all these samples (Fig. 6). The results show that the initial potential of the discharge plateau significantly decreases before the doping content approaches saturation (Fig. S7a and b†), further confirming that the energy of the Ti^4+^/Ti^3+^ redox couple in spinel Li_4_Ti_5_O_12_ could be changed by Al-doping. Also, we may see that the charging potential increment arising from different discharging cutoffs (Fig. 6b–h) shows the same dependence on the doping content (Fig. S7c†), suggesting that the memory effect of Al-doped Li_4_Ti_5_O_12_ can be tailored by changing the doping level. Although a small amount of impurities, *e.g.*, rutile TiO_2_, could be introduced by a high level of doping, their effect on the memory effect is insignificant.

## Conclusions

In this study, we show the Al-doping-induced memory effect in a spinel Li_4_Ti_5_O_12_ anode for LIBs. For the Al-doped Li_4_Ti_5_O_12_, the electrochemical kinetics, which can be evaluated by the overpotential, can be altered by changing the discharging cutoff. In a special memory-writing cycle, multiple discharging cutoffs can be recorded in a discharged electrode. In the following charge process, this information can be read as a stepped charging curve, which is indicative of the memory effect. The memory effect of Al-doped Li_4_Ti_5_O_12_ could be rationalized on the basis of the particle-by-particle model. Besides, the memory effect can be tailored by changing the doping level in Al-doped Li_4_Ti_5_O_12_. Our discovery demonstrates that the widely adopted element doping strategy is capable of triggering the memory effect in LIBs, which should be taken into account in industrial battery design.

## Supplementary Material

Supplementary information
