# Supplementary material for: Doping-induced memory effect in Li-ion batteries: the case of Al-doped Li4Ti5O12
Source: Chem Sci. 2015 Apr 17;6(7):4066–70. doi: 10.1039/c5sc00429b (PMC5497270; doi:10.1039/c5sc00429b)
Supplement: Supplementary file 1 [file SC-006-C5SC00429B-s001.pdf]

Electronic Supplementary Material (ESI) for Chemical Science.
This journal is © The Royal Society of Chemistry 2015

Supporting Information

Doping-induced memory effect in Li-ion battery: the case of Al-doped $Li_4Ti_5O_{12}$

*De Li, Yang Sun, Xizheng Liu, Ruwen Peng and Haoshen Zhou**

Supporting Information contains Fig. S1-7.

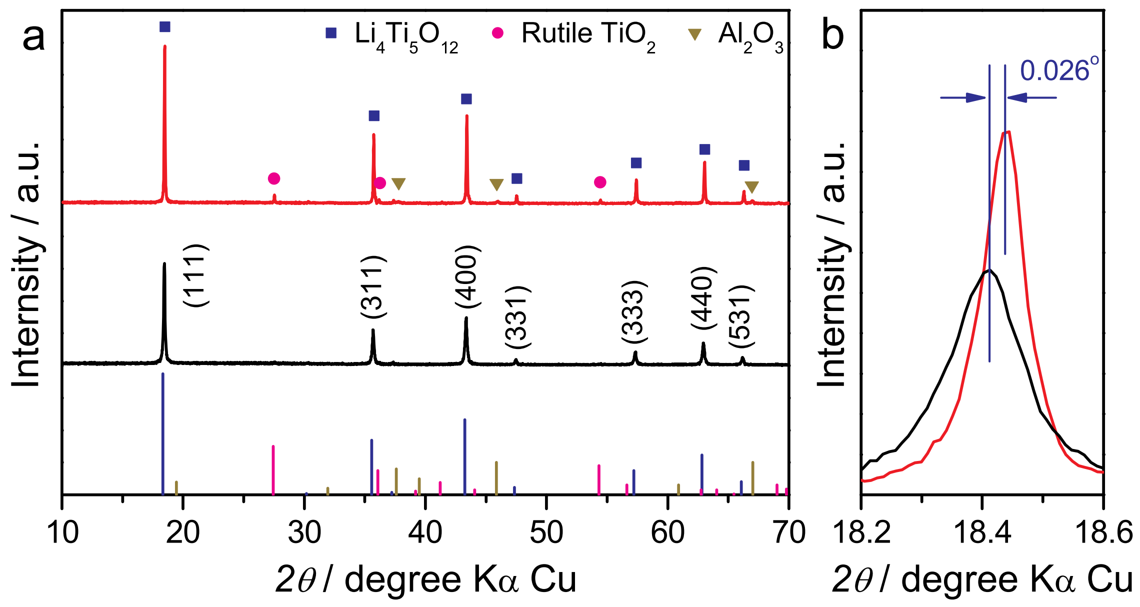

Fig. S1 (a) XRD patterns of pristine $Li_4Ti_5O_{12}$ (LTO, black curve) and Al-doped $Li_4Ti_5O_{12}$ (ALTO, red curve). (b) Enlarged XRD patterns of (111) peaks of LTO and ALTO.

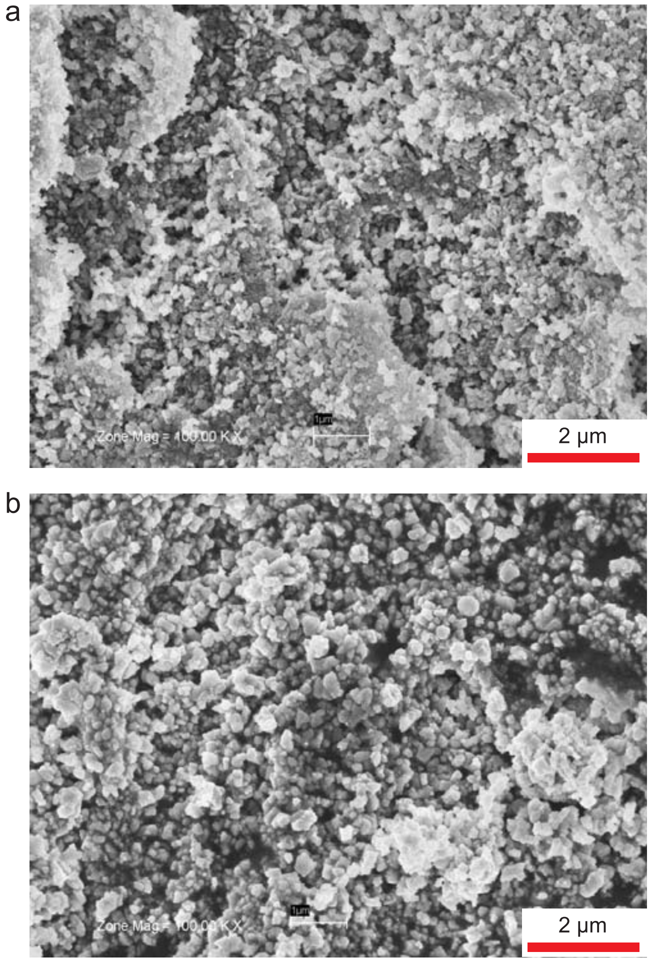

Fig. S2 SEM images of (a) pistine $Li_4Ti_5O_{12}$ (LTO) and (b) Al-doped $Li_4Ti_5O_{12}$ (ALTO). LTO is an assembly of nano-crystallites, and the primary nano-crystallites grow up and agglomerate in a certain extent in ALTO.

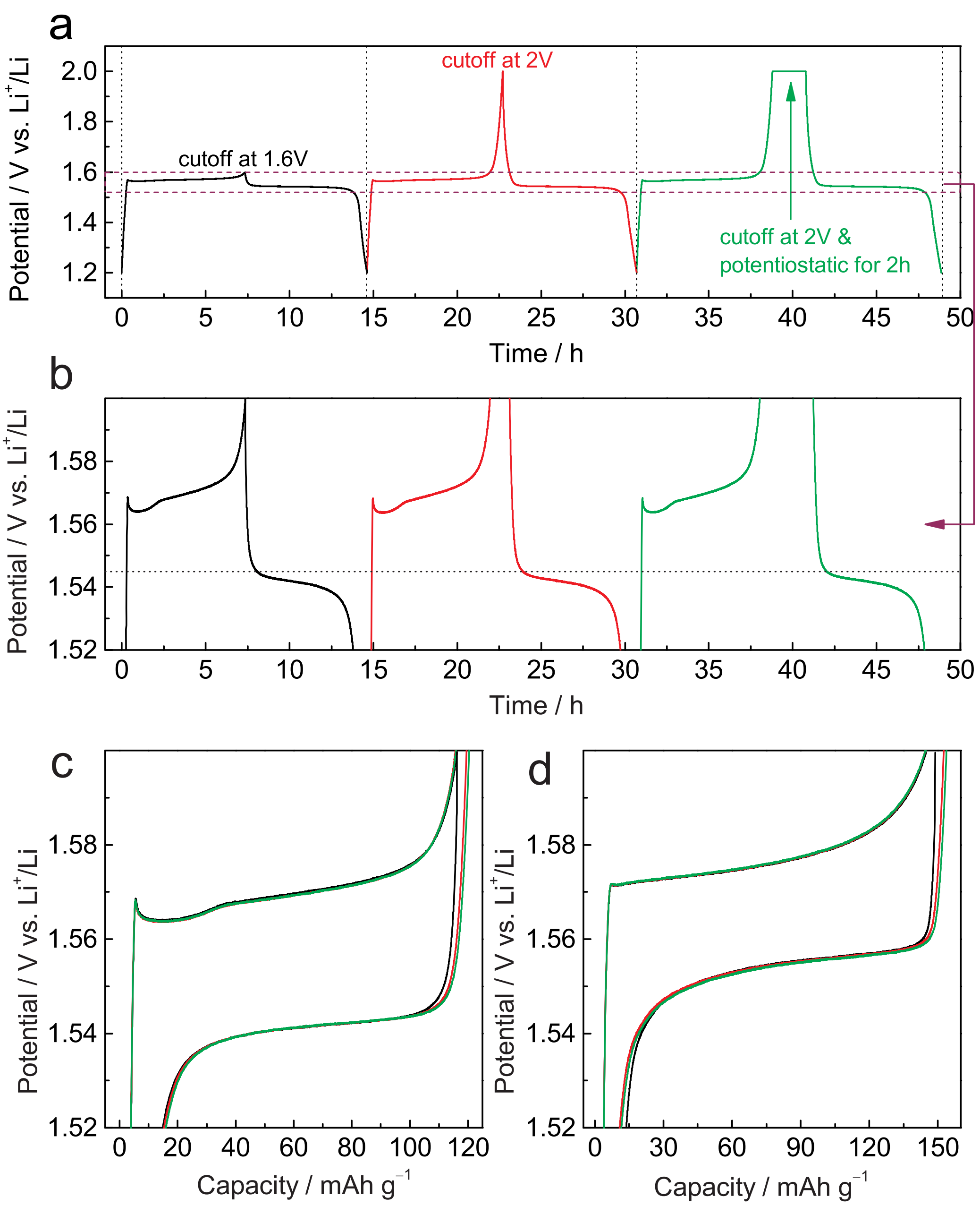

Fig. S3 Electrochemical dependence on the charging cutoff in Al-doped $Li_4Ti_5O_{12}$ (ALTO). (a) A sequence of three cycles: (1) charge to 1.6 V and full discharge; (2) charge to 2.0 V and full discharge; (3) charge to 2.0 V and potentiostatic for 2h, and full discharge. (b) Enlarged view between 1.52 and 1.60 V. (c) The charge/discharge curves in these three cycles. (d) The charge/discharge curves in the same three cycles of pristine $Li_4Ti_5O_{12}$ (LTO). The charge/discharge current rate is 0.1C.

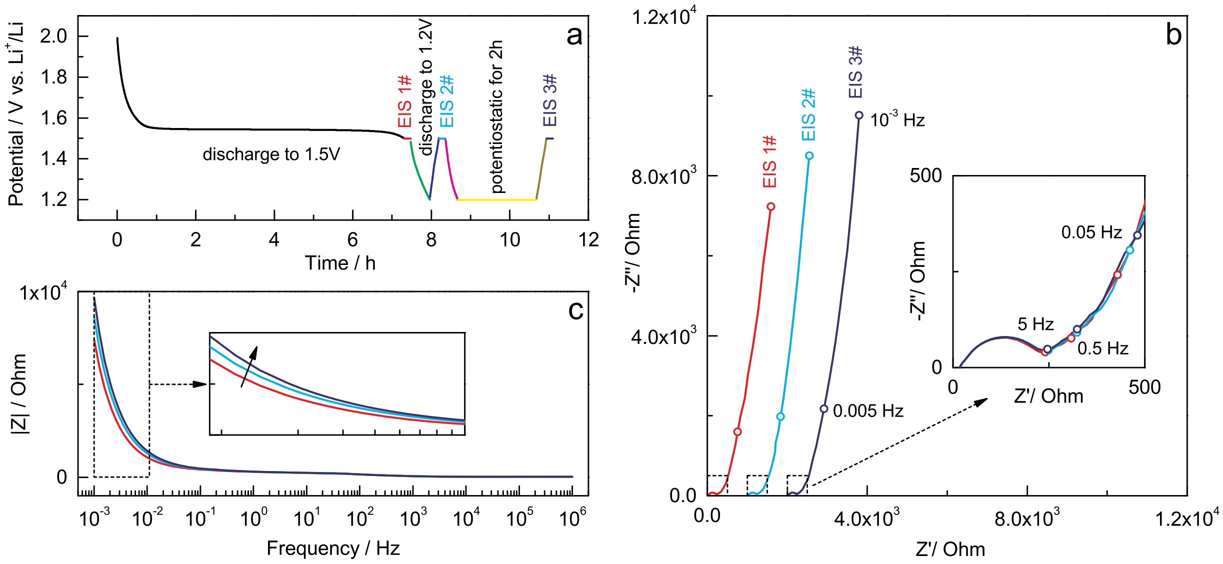

Fig. S4 EIS spectra for different discharging cutoffs in Al-doped $Li_4Ti_5O_{12}$ (ALTO). (a) An electrochemical sequence: discharge to 1.5 V and potentiostatic for 10 min, EIS measurement at 1.5 V (EIS 1#); discharge to 1.2 V, charge to 1.5 V and potentiostatic for 10 min, EIS measurement at 1.5 V (EIS 2#); discharge to 1.2 V and potentiostatic for 2h, charge to 1.5 V and potentiostatic for 10 min, EIS measurement at 1.5 V (EIS 3#). (b) Corresponding EIS results from 10^6 Hz to 10^{-3} Hz where three spectra were separated by offsets, and the enlarged high frequency region in the inset. (c) Magnitude plot of three EIS spectra. Here, EIS measurements were performed using a Solartron Analytical 1287 Electrochemical interface with a model 1255b Impedance Analyzer.

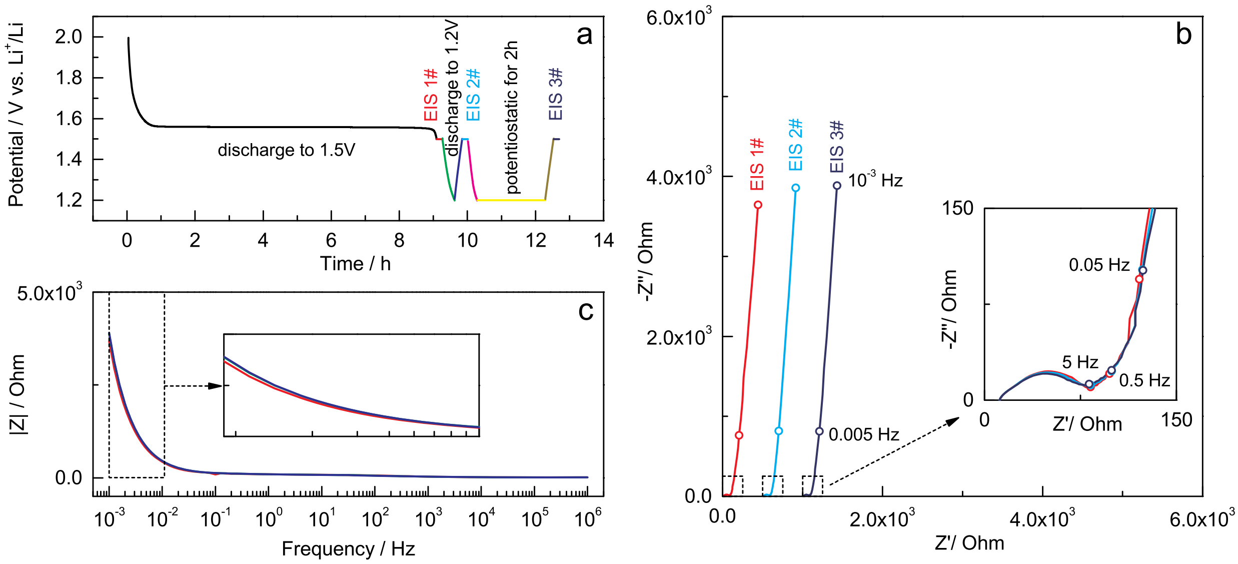

Fig. S5 EIS spectra for different discharging cutoffs in pristine $Li_4Ti_5O_{12}$ (LTO). (a) An electrochemical sequence: discharge to 1.5 V and potentiostatic for 10 min, EIS measurement at 1.5 V (EIS 1#); discharge to 1.2 V, charge to 1.5 V and potentiostatic for 10 min, EIS measurement at 1.5 V (EIS 2#); discharge to 1.2 V and potentiostatic for 2h, charge to 1.5 V and potentiostatic for 10 min, EIS measurement at 1.5 V (EIS 3#). (b) Corresponding EIS results from 10^6 Hz to 10^{-3} Hz where three spectra were separated by offsets, and the enlarged high frequency region in the inset. (c) Magnitude plot of three EIS spectra.

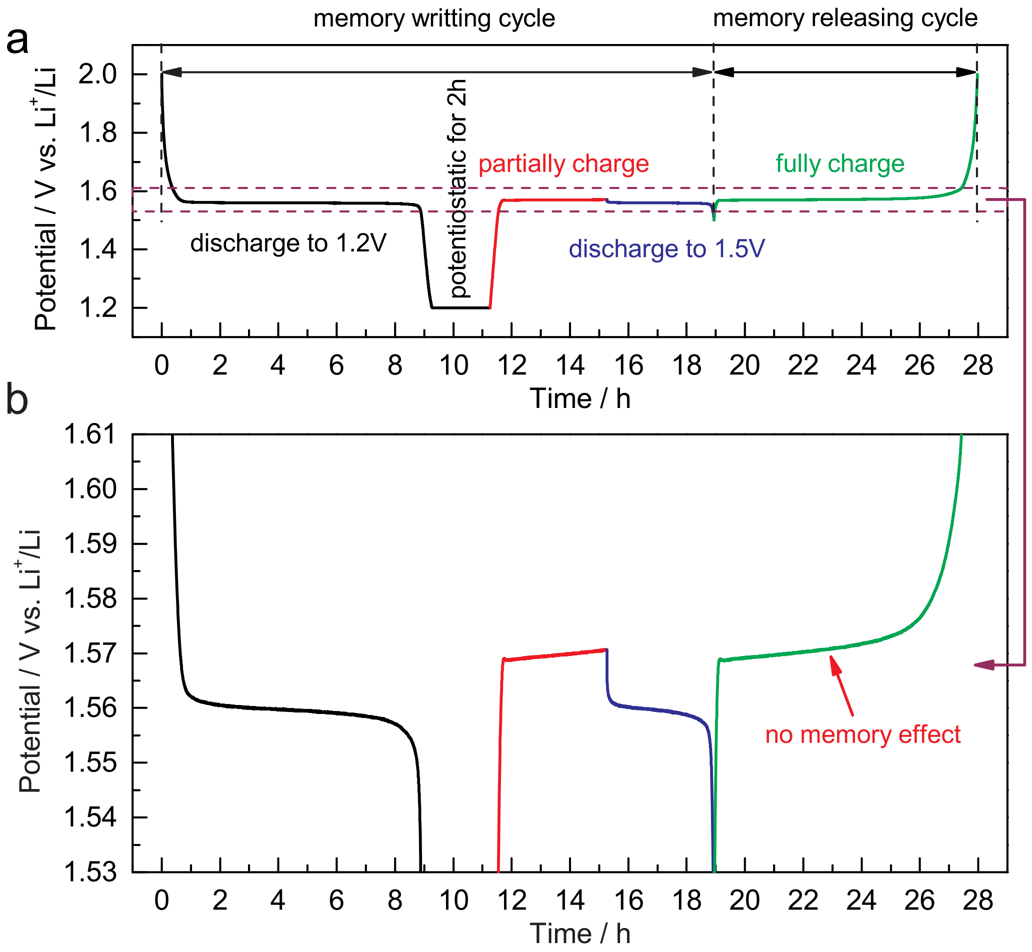

Fig. S6 Demonstration of no memory effect in pristine $Li_4Ti_5O_{12}$ (LTO). (a) Memory-writing cycle: discharge to 1.2 V and potentiostatic for 2h (black), partially charge for 4h (red), and discharge to 1.5 V (blue); m emory-releasing cycle: full charge to 2.0 V (green). The current rate is 0.1C. (b) Enlarged view between 1.53 and 1.61 V.

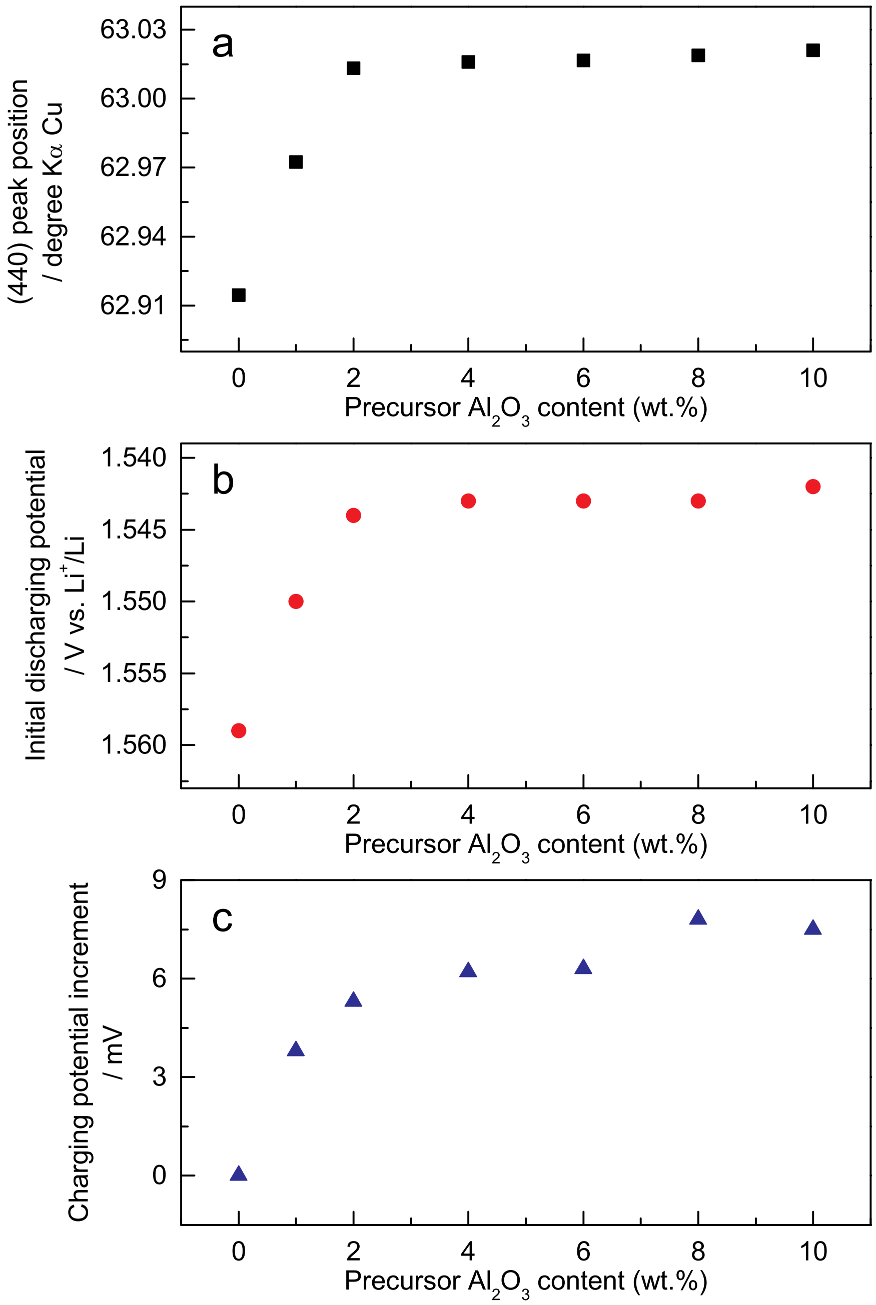

Fig. S7 The dependence of (a) (440) peak position from Fig. 5, (b) in itial discharging potential and (c) charging potential increment from Fig. 6, on the precursor Al_2O_3 content in a series of Al-doped $Li_4Ti_5O_{12}$.
